# A Hyperbolic Graph Neural Network Model with Contrastive Learning for Rating–Review Recommendation

**DOI:** 10.3390/e27080886

**Published:** 2025-08-21

**Authors:** Shuyun Fang, Junling Wang, Fukun Chen

**Affiliations:** 1 School of Software and Big Data Technology, Dalian Neusoft University of Information, Dalian 116023, China; 2 School of Computer and Artificial Intelligence, Liaoning Normal University, Dalian 116029, China

**Keywords:** hyperbolic graph neural network, contrastive learning, dual-graph construction, data sparsity

## Abstract

In recommender systems research, the data sparsity problem has driven the development of hybrid recommendation algorithms integrating multimodal information and the application of graph neural networks (GNNs). However, conventional GNNs relying on homogeneous Euclidean embeddings fail to effectively model the non-Euclidean geometric manifold structures prevalent in real-world scenarios, consequently constraining the representation capacity for heterogeneous interaction patterns and compromising recommendation accuracy. As a consequence, the representation capability for heterogeneous interaction patterns is restricted, thereby affecting the overall representational power and recommendation accuracy of the models. In this paper, we propose a hyperbolic graph neural network model with contrastive learning for rating–review recommendation, implementing a dual-graph construction strategy. First, it constructs a review-aware graph to integrate rich semantic information from reviews, thus enhancing the recommendation system’s context awareness. Second, it builds a user–item interaction graph to capture user preferences and item characteristics. The hyperbolic graph neural network architecture enables joint learning of high-order features from these two graphs, effectively avoiding the embedding distortion problem commonly associated with high-order feature learning. Furthermore, through contrastive learning in hyperbolic space, the model effectively leverages review information and user–item interaction data to enhance recommendation system performance. Experimental results demonstrate that the proposed algorithm achieves excellent performance on multiple real-world datasets, significantly improving recommendation accuracy.

## 1. Introduction

With the rapid advancement of Internet technology, information resources have experienced exponential growth, making the precise extraction of user demand information from massive datasets a critical challenge in the field of information retrieval. Traditional recommender systems primarily rely on collaborative filtering and content-based recommendation techniques. Although these approaches have achieved remarkable success in specific scenarios, they face significant challenges in deeply modeling user interests: Firstly, the explosive increase in the number of users and items accentuates the sparsity issue of rating matrices; secondly, sole reliance on numerical rating data hampers the development of recommendation models with semantic interpretability. Recent breakthroughs in deep learning have offered novel solutions for recommender systems. Notably, graph neural networks (GNNs) demonstrate the capability to effectively model high-order connectivity within user–item interaction graphs, thereby capturing implicit feature representations that are difficult to extract using traditional methods. Text-based modeling, a widely adopted recommendation paradigm, leverages textual information such as reviews, summaries, and abstracts, together with neural techniques, to distill essential document semantics. By augmenting sparse rating data with rich textual cues, this approach improves recommendation quality and interpretability [1,2]. Graph neural networks (GNNs) further unify heterogeneous user–item interactions into a high-order graph structure, capturing complex relations and multi-hop dependencies to enhance personalization and accuracy [3,4]. Recent efforts, exemplified by Zhang et al.’s [5] multi-attribute bipartite graph convolutional network, jointly encode reviews, ratings, and interaction signals, yielding significant gains in rating prediction.

Recommendation algorithms integrating review texts and graph neural networks (GNNs) can effectively leverage graph-structured data to capture complex user preferences and partial item characteristics. However, in recommender systems, the inherent sparsity of user–item interactions hinders the learning of high-quality representations for users and items. Additionally, while reviews contain rich semantic information, existing methods often inadequately integrate such multimodal data. To enhance model learning capability, introducing contrastive learning tasks has proven effective [6,7,8]. Furthermore, Jiang et al. [9] introduced the MMGCL method, which fuses review information with GNNs via multi-view graph-contrastive learning and meta-knowledge enhancement. This significantly improves the model’s generalization capacity and robustness.

However, prevailing graph neural networks for recommendation commonly embed user–item interactions and review information in Euclidean space, which exposes two critical bottlenecks in real-world scenarios. First, the Euclidean distance metric and inner-product operations struggle to characterize power-law distributions and hierarchical semantics, leading to embedding distortion and limited expressiveness. Second, the fine-grained sentiment and contextual signals inherent in review text are typically compressed into low-dimensional vectors under the Euclidean paradigm, causing both the curse of dimensionality and semantic collapse, and thus failing to excavate users’ deep-seated preferences. To address these issues, we propose a hyperbolic graph-contrastive recommendation model that jointly leverages ratings and reviews. The core motivation is to simultaneously enhance expressiveness and robustness. On the one hand, the exponential expansion property of hyperbolic geometry naturally accommodates the hierarchical structures exhibited by user interests, product categories, and review topics, enabling accurate encoding of complex relations at substantially lower dimensions, thereby mitigating distortion and reducing storage and computational costs. Meanwhile, hyperbolic distance amplifies subtle discrepancies between “semantically close but structurally distant” and “structurally close but semantically distant” pairs, allowing the model to sensitively distinguish nuanced sentiment gradients in reviews. On the other hand, to suppress redundancy and noise in reviews, we introduce a contrastive learning paradigm. Specifically, we separately encode a “rating-graph view” and a “review-graph view,” and then construct positive and negative sample pairs. By maximizing the consistency of the same node and minimizing the similarity between different nodes within the hyperbolic space, irrelevant information is naturally filtered out. This self-supervised signal is particularly critical under data-sparse or long-tail scenarios, as it does not rely on explicit interactions but leverages semantic similarities among reviews to generate reliable initial representations for cold-start items, thus alleviating popularity bias. We consists of three parts: review text modeling, hyperbolic GNN learning module, and contrastive learning module. The main contributions of this work can be summarized as follows:

(1) We propose a dual-graph strategy: A review-aware graph, in which users and items constitute nodes and review texts serve as edges, captures fine-grained user opinions and item descriptions, whereas a user–item graph, with rating scores as edges, encodes macro-level interaction strengths. By jointly learning over these complementary views, the model explicitly disentangles and fuses textual semantics with numerical preferences, thereby learning effective user and item representations from the complex user–item interaction patterns.
(2) We introduce a hyperbolic graph neural network framework that unifies hyperbolic embeddings and graph neural networks within a single representation space. By jointly performing hierarchical learning and structured feature extraction in hyperbolic space, the framework markedly enhances the model’s adaptability to the global properties of interaction data and substantially improves its capacity to represent complex graph structures.
(3) We introduce a contrastive learning framework to optimize the representations of users and items across different views. By performing contrastive learning tasks in hyperbolic space, we enhance the discriminative power of user and item representations. This process refines the quality of representations in hyperbolic graph space, enabling the model to capture more nuanced and complex patterns in the data.

## 2. Related Work

### 2.1. Review-Based Recommendation Models

Review texts reflect user preferences and item features, making them rich in semantic information. In recommendation systems, this richness helps address rating sparsity and enhances model interpretability. The TARMF model, proposed by Lu et al. [10], employs GRU with attention mechanisms to model user and item reviews and combines matrix factorization for recommendation tasks. Cataldo Musto et al. [1] showed that review texts can make recommendations more transparent using text summarization and sentiment analysis. Ren et al. [11] proposed the SGDN model, which uses user review data as auxiliary information. It models user–item interactions based on latent factors like quality, price, and appearance to alleviate data sparsity. Shuai et al. [12] proposed the RGCL recommendation model based on a review-aware graph. RGCL builds a user–item graph enhanced by review features. Each edge feature comprises user–item ratings and corresponding review semantics. This design enables precise learning of neighbor node weights for better user and item representation. Wang et al. [13] proposed a method where all historical reviews of users or items are used as input for pre-trained language models to predict ratings based on reviews. Bo Kong et al. [14] constructed a user–item review graph structure and combined it with GNNs and attention mechanisms. This methodology accurately captures semantic information within reviews and user preferences. Furthermore, Liu et al. [15] innovatively utilized GNNs to meticulously extract aspect performance-aware information from user reviews. They constructed a hypergraph and designed an aspect-aware hypergraph aggregation method to learn item performances across different aspects. CGRC [16] integrates masked graph auto-encoder structures and multimodal content. It directly incorporates interaction-based, high-order connectivity. This effectively tackles the challenge faced by traditional graph convolution methods in cold-start item recommendations, where leveraging high-order collaborative signals is difficult. Li et al. [17] propose the RNS model, which incorporates review semantics into collaborative filtering by leveraging aspect-aware representations to encode long-term preferences and a hierarchical attention-over-attention mechanism to capture short-term patterns, thereby addressing the limitations of conventional sequential recommenders that neglect review semantics and inadequately model the joint dynamics of long- and short-term preferences. Zhang et al. [18] introduce the FineRec framework that employs a large language model to extract attribute–opinion pairs from reviews and constructs attribute-specific graphs, enabling fine-grained user preference and item characterization through a diversity-aware convolution and an interaction-driven fusion mechanism, thus overcoming the coarse modeling inherent in existing sequential recommendation approaches.

### 2.2. Graph Neural Network-Based Recommendation Models

Graph neural networks (GNNs) [19,20] are deep learning methods for graph domain analysis. By message propagation between nodes, GNNs capture dependencies on graphs. In recommendation systems, most information, such as knowledge graphs and user–item bipartite graphs, has a graph structure. GNNs can capture complex and indirect interactions by passing and updating information layer by layer, and it can also effectively integrate the information contained in the edges connecting the nodes. Graph neural network-based recommender systems [21,22,23,24], including LightGCN [25], RGCL [12], CECL [26], and DeHier [27], offer new solutions for addressing information overload. He et al. found that in Graph Convolutional Networks (GCNs), operations such as feature transformation and the use of nonlinear activation functions hardlyhave any impact on the performance of collaborative filtering. A simplified GCN model retaining only essential components was then proposed. RGCL integrates reviews into graph learning and uses GNNs to capture high-order relationships in graphs. CECL introduces category information as a direct supervisory signal into recommendation systems. This helps obtain high-quality user–item interactions. It uses GNNs to grasp complex user–item relationships and boost system performance. DeHier employs hierarchical GNN layers to propagate diverse user interests from historical to current sessions and to learn short-term user preferences. Similarly, Yang et al. [28] proposed the GANCL framework, a novel GNN approach combining Graph Attention Networks (GATs) and contrastive learning. It integrates temporal review features and rating data into a user–item bipartite graph to extract features that better capture users’ dynamic preferences and uncover deeper user–item associations. Nikorn Kannikaklang et al. [29] propose the Bidirectional Graph Convolutional Attention Network, which captures both long- and short-term preferences through bidirectional modeling and a gated fusion mechanism, thereby alleviating the performance bottleneck in sequential recommendation caused by unidirectional information loss and insufficient coupling of temporal preferences.

### 2.3. Hyperbolic Recommendation Models

In recommendation systems, most interaction data shows non-Euclidean properties, like power-law distributions. Mapping such data to Euclidean space causes embedding distortion and degrades recommendation performance. Hyperbolic space, with its exponentially growing continuous tree-like structure, can naturally represent hierarchical or scale-free network data, reducing embedding distortions. Based on this, a series of innovative methods have emerged. The HNCR model [30], based on hyperbolic geometry, incorporates neighborhood sets into the recommendation process through a deep learning framework. It learns high-quality user and item representations. Qiyao Ma et al. [31] proposed the HARec model, which uses hyperbolic geometry for hierarchical modeling and employs a hyperbolic hierarchical tree structure to learn user–item representations. Zhang et al. [32] addressed the need for improved representation learning quality in personalized tag recommendation by proposing a combination of graph neural networks and hyperbolic embedding to enhance the quality of user, item, and tag representations. Yoonhyuk Choi et al. [33] embedded review texts into hyperbolic space to prevent hierarchy destruction caused by domain alignment techniques. used hyperbolic geometry to better capture complex logical relationships in collaborative knowledge graphs. Hu et al. [34] propose the hyperbolic collaborative knowledge graph learning model HCKGL, which incorporates relation-aware curvature graph attention and multi-level contrastive learning in hyperbolic space, thereby addressing the limitations of existing Euclidean approaches in capturing hierarchical structures and complex relational logic, as well as their inability to precisely assess neighbor contributions.

## 3. Methods and Models

In this section, the proposed recommendation model with hyperbolic embedding and contrastive learning based on graph neural networks for integrating ratings and reviews is described in detail. Initially, a review-aware graph and a user–item graph were constructed, with nodes representing users and items, and edges denoting their interactions. Next, a graph neural network model combining attention mechanisms and hyperbolic space was employed. The attention mechanisms identified key information in reviews, while hyperbolic space better represented complex node relationships. Finally, contrastive learning was introduced to construct positive and negative sample pairs. This helped the model distinguish between related and unrelated user–item pairs, refining user and item representations in the hyperbolic graph space. Combining LightGCN [25] with hyperbolic space constructs a hyperbolic graph neural network. Hyperbolic space, with its negative curvature, is ideal for representing hierarchical data.

### 3.1. Review-Aware Graph and a User–Item Graph

This research aims to mine review information to capture users’ personalized preferences accurately. To this end, it adopts a dual-graph construction approach. This approach can fully capture the subtle differences in user preferences and the multi-dimensional attributes of item features. First, organize the existing data into a natural user–item bipartite graph structure with user rating information, providing a clear data foundation for subsequent analysis. Second, construct a review-aware graph learning model. This model can more effectively mine collaborative signals among users and potential correlations in review texts, thus enhancing recommendation system performance and accuracy. First, initialize all input parameters, including node embedding vectors and edge features, and define matrices U(|U|=M) and V(|V|=N) to represent the sets of users and items. Within this framework, specific entities, user *i* and item *j*, are represented as $e_{i}\in U$ and $e_{j}\in V$, respectively. The rating records are represented as a user–item rating matrix $R\in R^{M\times N}$,where $r_{ij}$∈{1,2,3,4,5} denotes the rating given by user *i* and item *j*, and $e_{ij}$$\in R^{d}$ denotes the corresponding review.

#### 3.1.1. Initialize the User–Item Graph

**Figure 1:**
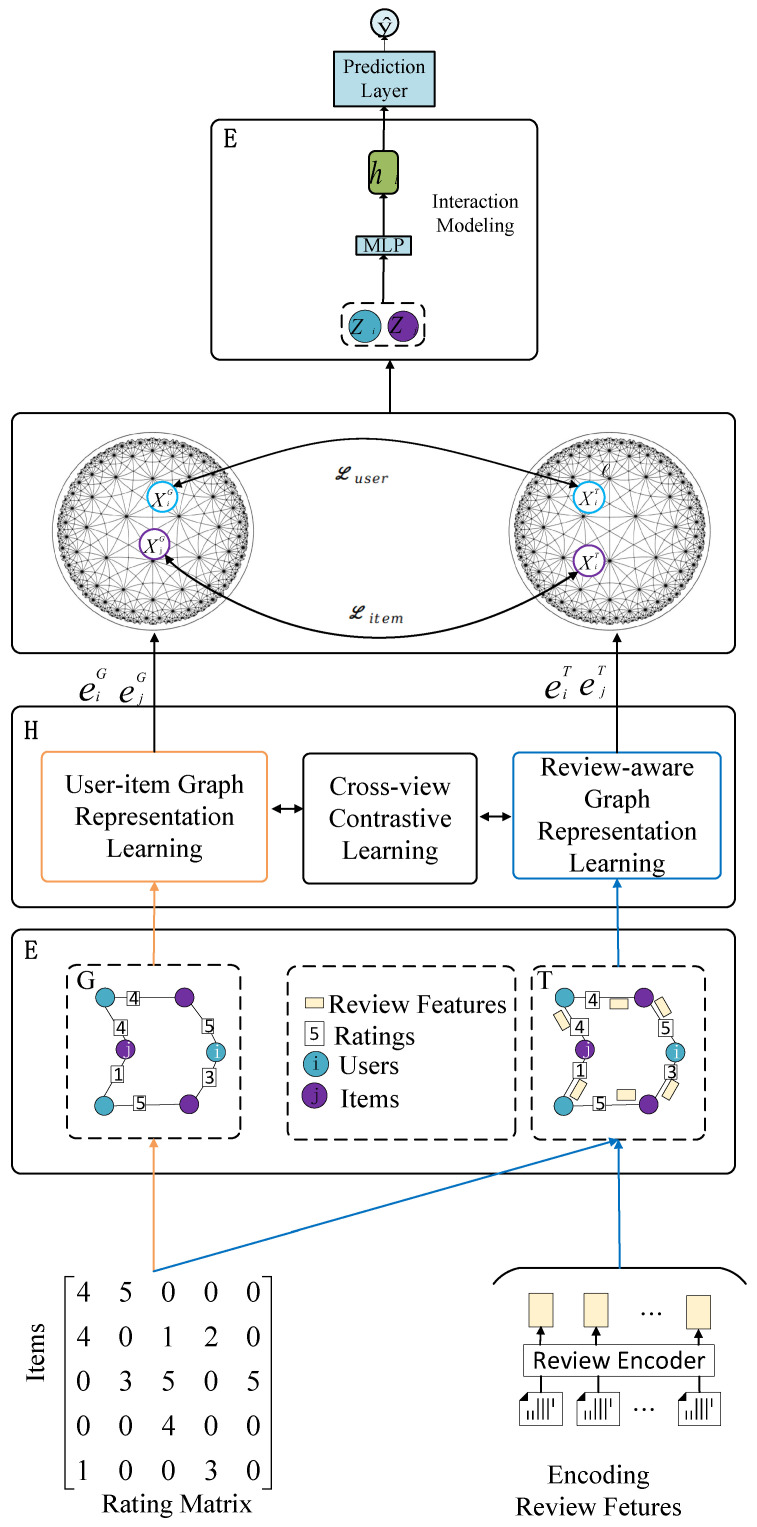
Overview of the model.

**Figure 2:**
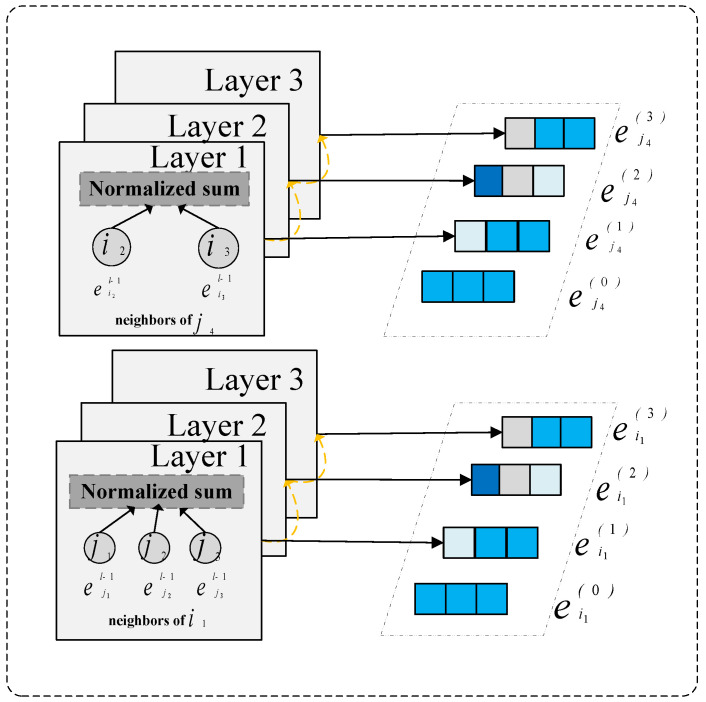
Overview of the model.

When constructing the user–item graph *G*, graph theory methods are employed. Users and items are abstracted as graph nodes, and user–item interactions serve as edges to form connections. The sets of user and item entities are represented as $U^{G}$ and $V^{G}$. In this framework, $e_{i}^{G}$ and $e_{j}^{G}$ denote specific user and item entities. Edges represent user ratings for items, quantified as $r_{ij}$, establishing a quantitative user–item relationship. This forms a bipartite graph where users and items are disjoint vertex sets, and edges connect vertices from different sets, reflecting user–item interactions. (See Figure 1 and Figure 2).

#### 3.1.2. Initialize the Review-Aware Graph

In this step, the review-aware graph T is constructed. User and item matrix embeddings are $U^{T}$ and $V^{T}$. Specifically, $e_{i}^{T}$ and $e_{j}^{T}$ denote users and items. The user rating $r_{ij}$ is used as an edge feature to capture semantic differences in ratings. To generate embeddings $e_{ij}$ that accurately represent the reviews of user *i* and item *j*, the BERT-Whitening [35] algorithm is employed. It captures deep semantic features of text data and enhances embedding quality through whitening, providing richer and more precise representations for subsequent graph learning tasks.

### 3.2. Hyperbolic Embedding

#### Interactive Graph Generation and Representation

Hyperbolic space is a unique continuous geometric space marked by its negative curvature present in any infinitesimal region, which remains constant across the vast expanse of the space. This intrinsic property enables hyperbolic space to serve as an optimal framework for representing power-law-distributed data structures, such as tree-like structures. Its ability to efficiently and accurately capture the intrinsic hierarchical relationships and complex distribution patterns within data makes it a superior choice for such representations. Due to its complex geometry, hyperbolic space is hard to visualize intuitively. To facilitate research and application, academia often describes it using five classical isometric models. The Poincaré Ball model and the Lorentz model are particularly popular. The Poincaré Ball model can intuitively display the unique shape and properties of hyperbolic space; the Lorentz model is convenient for theoretical analysis and mathematical calculations, making complex processes simpler. This study adopts the Poincaré ball model, whose exponential and logarithmic mappings are as follows:

(1) $$\left\{\begin{array}{l} {\mathrm{Exp}}_{o}\left(s\right)=\tanh\left(∥s∥\right)\frac{s}{∥s∥}\\ \log_{o}\left(y\right)=\mathrm{arctanh}\left(∥y∥\right)\frac{y}{∥y∥} \end{array}\right.$$

**Figure 3:**
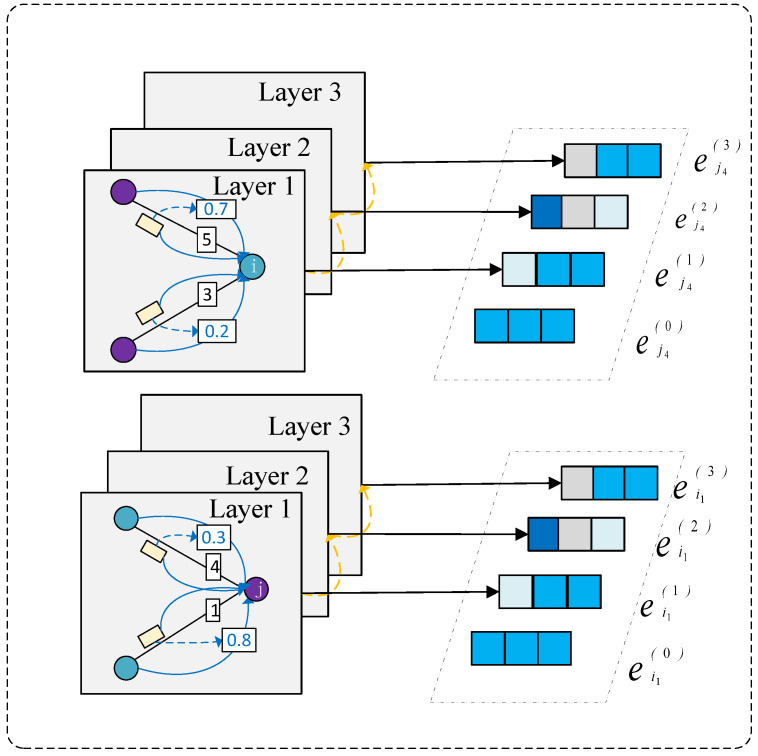
Overview of the model.

In the Poincaré ball model, ${Exp}_{o}\left(\cdot \right)$ and $log_{o}\left(\cdot \right)$ denote the exponential and logarithmic mappings, respectively, with O indicating the origin. (See Figure 3).

### 3.3. User–Item Graph Representation Learning

#### 3.3.1. Hyperbolic Graph Convolutional Layer

In hyperbolic space, graph convolution operations need to account for the geometric properties of hyperbolic space. The embedding update formula for the l−th layer is as follows:

(2) $$\left\{\begin{array}{c} X_{e_{i}^{G}}^{\left(l\right)}={\mathrm{Exp}}_{o}(\sum_{j\in N(i)}\frac{r_{ij}}{\sqrt{d_{i}d_{j}}}e_{j}^{G^{\left(l-1\right)}})\\ X_{e_{j}^{G}}^{\left(l\right)}=\mathrm{Exp}o(\sum_{i\in N(j)}\frac{r_{ij}}{\sqrt{d_{j}d_{i}}}e_{i}^{G^{\left(l-1\right)}}) \end{array}\right.$$

Here, N(i) denotes the set of neighbors for node *i*, and N(j) denotes the set of neighbors for node *j*. To incorporate the rating weights between nodes *i* and *j* as edge information, the sum of neighbor rating weights is used instead of the number of neighbors for normalization. $d_{i}$ and $d_{j}$ represent the sums of neighbor rating weights for nodes *i* and *j*, respectively.

#### 3.3.2. Multi-Layer Embedding Aggregation

Hyperbolic graph neural networks aggregate embeddings across layers through a weighted sum to obtain the final representation:

(3) $$\left\{\begin{array}{c} x_{i}^{G}=\sum_{i=0}^{L}\alpha_{i}X_{e_{i}}^{\left(1\right)}\\ x_{j}^{G}=\sum_{i=0}^{L}\alpha_{i}X_{e_{j}}^{\left(1\right)} \end{array}\right.$$

Here, $\alpha_{l}$ is the weight for the l−th layer, which can typically be set as $\alpha_{l}=1/\left(L+1\right)$.

### 3.4. Review-Aware Graph Representation Learning

In this stage, by examining theratings and reviews attached to the edges, we measure the potential impact of the surrounding related nodes and past reviews on the central node. This process allows the model to capture complex interactions between nodes, thereby providing rich contextual information for node embedding learning. Subsequently, messages received from neighboring nodes are aggregated to update the embedding representation of the central node. This step is crucial for capturing collaborative signals within the local neighborhood.

#### 3.4.1. Review-Aware Message Passing

In recommendation systems, user–item interactions typically encompass two key features: ratings and review semantics. Compared to singular numerical ratings, textual reviews possess unique advantages due to their rich, fine-grained semantic information. The detailed semantics embedded in reviews can precisely reveal user preferences and item characteristics, which is crucial for learning accurate user and item embeddings. By deeply analyzing these fine-grained semantics, models can gain a more profound and precise understanding of the complex and subtle relationships between users and items. This deep understanding enables models to accurately identify user preferences for different items, thereby further optimizing the mutual influence weights between users and items. In hyperbolic space, message passing needs to consider hyperbolic distance and hyperbolic attention mechanisms. For user *i* and item *j*, along with their review information $e_{i}j$, message passing in hyperbolic space can be defined as:

(4) $$\left\{\begin{array}{c} m_{j\to i}^{\left(l-1\right)}={\mathrm{Exp}}_{o}\left(W_{j}e_{j}^{T(l-1)}+W_{e}e_{ij}^{\left(l-1\right)}\right)\\ m_{i\to j}^{\left(l-1\right)}={\mathrm{Exp}}_{o}\left(W_{i}e_{i}^{T(l-1)}+W_{e}e_{ij}^{\left(l-1\right)}\right) \end{array}\right.$$

where $W_{j}$, $W_{i}$, and $W_{e}$ are trainable weight matrices.

#### 3.4.2. Review-Aware Graph Weighted Aggregation

In the context of graph-structured data, users and items are regarded as nodes. This study employs an aggregation mechanism that integrates all messages received by these nodes into a unified representation. Specifically, this process involves a systematic aggregation operation on the messages collected by each node, aiming to refine the embedding vectors of users and items by pooling information from their neighborhoods. This step is crucial within the framework of graph neural networks for capturing complex relationships between nodes and enhancing the quality of embedding vectors. The hyperbolic embeddings of user *i* and item *j* can be updated by aggregating information from neighboring nodes and then normalized to prevent the embedding norms from becoming too large and exceeding the Poincaré ball.

(5) $$\left\{\begin{array}{c} X_{e_{i}^{T}}^{\left(1\right)}=\sum_{j\in N(i)}\frac{\beta_{ij}}{\sqrt{D_{i}D_{j}}}\cdot m_{j\to i}^{\left(l-1\right)}\\ Y_{e_{i}^{T}}^{\left(1\right)}=\sum_{j\in N(j)}\frac{\beta_{ij}}{\sqrt{D_{i}D_{j}}}\cdot m_{i\to j}^{\left(l-1\right)} \end{array}\right.$$

Here, N(i) denotes the set of neighbors for node *i*, and N(j) denotes the set of neighbors for node *j*. $\beta_{ij}$ represents the combined weight of rating and review, while $D_{i}$ and $D_{j}$ are weighted degrees that balance active nodes.

(6) $$\left\{\begin{array}{c} \beta_{ij}=\mathrm{Softplus}\left(W_{\beta }^{T}\left[r_{ij}\cdot W_{r}\|e_{ij}\right]\right)\\ D_{i}=\sum_{j}\beta_{ij},\;D_{j}=\sum_{i}\beta_{ij} \end{array}\right.$$

Here, $W_{r}$ denotes the rating weight vector, $W_{\beta }$ is a learnable parameter, and $\mathrm{Softplus}\left(x\right)=\ln\left(1+e^{x}\right)$ ensures that the weights are non-negative.

#### 3.4.3. Multi-Layer Embedding Aggregation

Hyperbolic graph neural networks aggregate embeddings across layers through a weighted sum to obtain the final representation:

(7) $$\left\{\begin{array}{c} x_{i}^{T}=\sum_{i=0}^{L}\alpha_{i}X_{e_{i}}^{\left(1\right)}\\ x_{j}^{T}=\sum_{i=0}^{L}\alpha_{i}X_{e_{j}}^{\left(1\right)} \end{array}\right.$$

Here, $\alpha_{l}$ is the weight for the l−th layer, which can typically be set as $\alpha_{l}=1/\left(L+1\right)$.

### 3.5. Cross-View Contrastive Learning

**Figure 4:**
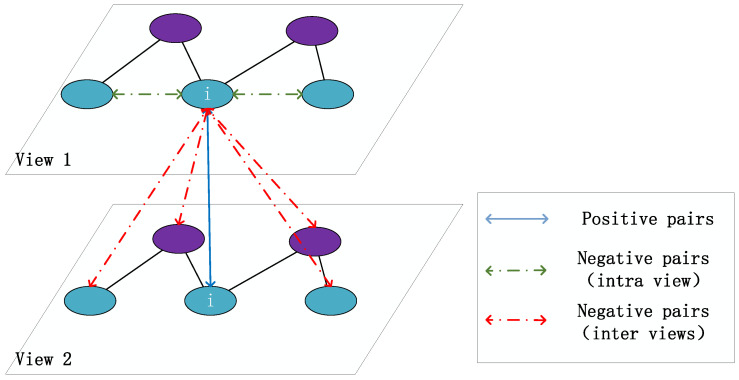
Cross-view contrastive learning.

Contrastive loss functions aim to enhance representations by pulling semantically similar samples closer together while pushing unrelated samples apart. (See Figure 4). The cross-view contrastive loss for users is as follows:

(8) $$\left\{\begin{array}{c} L_{user}=-\sum_{i}\log\frac{\mathrm{Exp}\left(\frac{{\mathrm{sim}}_{D}\left(X_{i}^{G},X_{i}^{T}\right)}{\tau }\right)}{\sum_{k}\mathrm{Exp}\left(\frac{{\mathrm{sim}}_{D}\left(X_{i}^{G},X_{k}^{T}\right)}{\tau }\right)}\cdot \frac{\gamma_{ij}\delta_{ij}}{\sqrt{D_{i}D_{j}}} \end{array}\right.$$

Here, $\gamma_{ij}$ is the rating weight, reflecting the user’s preference intensity for the item, $\delta_{ij}$ is the review weight, reflecting the importance of review semantics, and $D_{i}$ and $D_{j}$ are weighted degrees. Using hyperbolic distance $d_{D}$ as the similarity measure, we can obtain the following:

(9) $$\left\{\begin{array}{c} {\mathrm{sim}}_{D}\left(X_{i}^{G},X_{i}^{T}\right)=-\theta \cdot d_{D}\left(X_{i}^{G},X_{i}^{T}\right) \end{array}\right.$$

Here, θ>0 is a scaling factor, and $d_{D}$ denotes the Poincaré ball distance.

(10) $$\left\{\begin{array}{c} d_{D}\left(X_{i}^{G},X_{i}^{T}\right)=\mathrm{arcosh}(1+2\frac{∥X_{i}^{G}-X_{i}^{T}{∥}^{2}}{(1-∥X_{i}^{G}{∥}^{2})(1-∥X_{i}^{T}{∥}^{2})}) \end{array}\right.$$

Similarly, the cross-view contrastive loss for items is obtained as follows:

(11) $$L_{item}=-\sum_{j}\log\frac{\mathrm{Exp}\left(\frac{{\mathrm{sim}}_{D}\left(X_{i}^{G},X_{j}^{T}\right)}{\tau }\right)}{\sum_{k}\mathrm{Exp}\left(\frac{{\mathrm{sim}}_{D}\left(X_{j}^{G},X_{k}^{T}\right)}{\tau }\right)}\cdot \frac{\gamma_{ij}\delta_{ij}}{\sqrt{D_{i}D_{j}}}$$

### 3.6. Interaction Representation Modeling

Project it into Euclidean space through logarithmic mapping.

(12) $$\left\{\begin{array}{c} z_{i}^{G}={\mathrm{Log}}_{G}\left(X_{i}^{G}\right)\\ z_{j}^{G}={\mathrm{Log}}_{G}\left(X_{j}^{G}\right) \end{array}\right.$$

Similarly, $z_{i}^{G}={\mathrm{Log}}_{G}\left(X_{i}^{G}\right)$,$z_{j}^{G}={\mathrm{Log}}_{G}\left(X_{j}^{G}\right)$.

### 3.7. Rating Prediction

In hyperbolic space, rating prediction for users *i* and items *j* can be achieved through the inner product of their hyperbolic embeddings.

(13) $$\left\{\begin{array}{c} z_{i}=z_{i}^{G}\cdot z_{i}^{⊤}\\ z_{j}=z_{j}^{G}\cdot z_{j}^{⊤} \end{array}\right.$$

Here, · denotes the inner product operation in hyperbolic space. Concatenate the Euclidean vectors of users and items and input them into an MLP:

(14) $$h_{o}={\left[z_{i}\;\|\;z_{j}\right]}^{⊥}$$

After passing through an L-layer fully connected network:

(15) $$h_{l}=\sigma \left(W_{l}h_{l-1}+b_{l}\right)\;\left(l=1,2,\cdots ,L-1\right)$$

Here, σ is the activation function. The final rating prediction is

(16) $$\hat{y}=W_{L}h_{l-1}+b_{L}$$

Here, $W_{L}$ and $b_{L}$ represents learnable parameters.

### 3.8. Model Training and Loss Function

Rating prediction uses Mean Squared Error (MSE) as the loss function:

(17) $$L=\frac{1}{\left|D\right|}\sum_{(i,j,y)\in D}{\left(y-\hat{y}\right)}^{2}$$

Here, D denotes the training set data. The total loss function is

(18) $$L_{total}=\theta L+\delta {\left(L_{user}+L_{item}\right)}^{2}$$

Here, θ and δ are task balancing coefficients. In the hybrid optimization strategy, the optimization methods for rating prediction loss and hyperbolic contrastive loss are as follows: The Adam method is used for optimization. Specifically, the weight parameters W and bias parameters *b* of the recommendation model are updated via the Adam optimizer. The Adam optimizer combines the advantages of two methods. One is momentum, which helps the optimizer not be easily disturbed by small fluctuations when adjusting parameters. The other is adaptive learning rate, which allows the optimizer to automatically adjust the learning process according to different situations, effectively adjusting parameters to enable the recommendation model to better fit the training data and enhance recommendation accuracy. The optimization of hyperbolic parameters in the hyperbolic contrastive loss function is also carried out using the Adam optimizer. This optimization process consists of two steps. First, the mapping parameters $z_{i}^{G}$ and $z_{i}^{T}$ are updated in Euclidean space using the Adam optimizer. This step is similar to conventional Adam optimization, where the gradients are computed and the Adam update rules are applied to adjust the values of parameters $z_{i}^{G}$ and $z_{i}^{T}$ as follows:

(19) $$\left\{\begin{array}{c} \nabla_{X_{i}^{G},X_{i}^{T}}L_{user}=\frac{\partial L_{user}}{\partial z_{i}^{G}}\cdot \frac{\partial z_{i}^{G}}{\partial X_{i}^{G}}+\frac{\partial L_{user}}{\partial z_{i}^{T}}\cdot \frac{\partial z_{i}^{T}}{\partial X_{i}^{T}}\\ \nabla_{X_{j}^{G},X_{j}^{T}}L_{item}=\frac{\partial L_{item}}{\partial z_{j}^{G}}\cdot \frac{\partial z_{j}^{G}}{\partial X_{j}^{G}}+\frac{\partial L_{item}}{\partial z_{j}^{T}}\cdot \frac{\partial z_{j}^{T}}{\partial X_{j}^{T}} \end{array}\right.$$

This hybrid optimization strategy combines the efficiency of the Adam optimizer in Euclidean space with the properties of exponential mapping in hyperbolic space. It enables the model to effectively learn embedding representations in hyperbolic space while achieving good performance on recommendation tasks.

### 3.9. Model Optimization

In the framework, multi-layer aggregation is conducted across various modalities, with optimization achieved through cross-modality contrastive learning. Ultimately, the modality-independent representations for user *u*—specifically $z_{u}^{t}$ and $z_{u}^{k}$—are similarly derived, as are the modality-independent representations for item *v*—namely $z_{v}^{t}$ and $z_{v}^{k}$. By concatenating these representation forms, we obtain the final representations for both users and items. Subsequently, the relevance between the user and the item is assessed by computing the inner product of the vector corresponding to the target user with that of the candidate item, as elaborated below:

(20) $$\left\{\begin{array}{c} z_{u}^{*}=\left(z_{u}+z_{u}^{t}\right)\;\|\;z_{u}^{k}\\ z_{v}^{*}=\left(z_{v}+z_{v}^{t}\right)\;\|\;z_{v}^{k}\\ y_{uv}=z_{u}^{*T}z_{v}^{*} \end{array}\right.$$

To enhance the overall efficacy of the model, a multi-task training framework was employed to simultaneously tackle several interrelated tasks. This methodology allows the model to capitalize on knowledge across diverse learning objectives. By incorporating the recommendation task with self-supervised learning, the model gains advantages from both explicit labels and its capacity to autonomously discern patterns within large volumes of unlabeled data. In the context of knowledge-aware recommendation tasks, the BPR [36] loss function improves the efficacy of personalized recommendations by reconstructing users’ historical interaction data and positing that items with which users have engaged receive higher scores than those they have not interacted with. The specific calculation method is outlined as follows:

(21) $$L_{BPR}=\sum_{(u,v,v^{\prime })\in O}-ln\sigma \left({\hat{y}}_{uv}-{\hat{y}}_{uv^{\prime }}\right)$$

where $O=\left(u,v,v^{\prime }\right)|\left(u,v\right)\in O^{+},\left(u,v^{\prime }\right)\in O^{-}$ as a training dataset. $O^{+}$ represents the interactions that have previously occurred between users and items, while $O^{-}$ encompasses those items with which users have not yet interacted. By integrating cross-modality contrastive loss with BPR loss, we strive to optimize model parameters at both global and local levels to enhance learning effectiveness. The formulation of the optimization objective function is delineated as follows:

(22) $$L_{TK}=L_{BPR}+\alpha L_{CL}+\lambda {\left\|\;\Theta \;\right\|}_{2}^{2}$$

where Θ represents the collection of model parameters, α and λ are two hyperparameters controlling the ratio of contrast learning loss $L_{CL}$ and regularization loss $L_{2}$ in the loss function, respectively.

## 4. Experiments

In order to assess the efficacy of the algorithm introduced in this paper, experiments were carried out utilizing 5 real-world datasets. A selection of pertinent recommendation algorithms were chosen to serve as benchmark algorithms for comparison. These encompass traditional content-based recommendation algorithms, collaborative filtering-based recommendation algorithms, as well as existing graph neural network-based recommendation algorithms. The outcomes of the experiments reveal that the proposed hyperbolic graph neural network model with contrastive learning for rating–review recommendation surpasses the benchmark algorithms across multiple evaluation metrics. A thorough analysis of the experimental results was conducted, and the impact of varying parameter settings on algorithm performance was examined and discussed.

### 4.1. Datasets

In terms of experimental datasets, we selected multiple real-world datasets from different domains, such as user review data from *e*-commerce platforms and movie review data from film platforms. These datasets vary in scale and sparsity, allowing for a more comprehensive evaluation of the algorithm’s performance. (See Table 1).

### 4.2. Evaluation Metrics

Following established practices in prior studies [37,38], we adopt Mean Squared Error (MSE) as the evaluation metric for model performance, which is widely recognized for rating prediction tasks. To ensure the reliability of experimental results, all tests were independently repeated five times, with final results reported in the format of “mean ± standard deviation.” According to the criteria defined in the relevant literature [39,40], a performance improvement exceeding 1% is considered statistically significant in review-based recommendation systems.

### 4.3. Comparison Model

- SVD [41] breaks down the user–item rating matrix into simpler low-rank matrics to uncover hidden semantic features, addressing issues of sparsity, scalability, and overfitting in recommendation systems.
- NCF [42] introduces a neural network architecture to learn the nonlinear interactions between users and items, addressing the limitations of traditional collaborative filtering methods in capturing complex user preferences and item features.
- DeepCoNN [43] integrates review text information to address the limitations of traditional recommendation systems that rely solely on rating data, making it difficult to capture user preferences and item characteristics.
- NARRE [44] introduces an attention mechanism to model user reviews, automatically identifying the most influential review segments for rating prediction.
- DAML [45] uses rating information to guide review feature extraction and enhances rating prediction with review information, addressing the issue of insufficient integration of rating and review information in traditional recommendation systems.
- SDNet [37] incorporates external knowledge into recommendation systems efficiently through adversarial training, addressing the inefficiency and difficulty in integrating large-scale external knowledge in traditional recommendation systems.
- TransNets [46] learns the dynamic transformation relationships of user and item features, addressing the issue of overly static user preference and item characteristic representations in traditional recommendation systems that fail to capture dynamic interactions.
- GC-MC [47] represents user–item interaction data as a graph structure to address issues in recommendation systems.
- RMG [48] constructs a heterogeneous user–item review graph and uses a hierarchical attention mechanism to model both internal review information and multiple reviews for users/items.
- SSG [49] models user review information from three different perspectives—sets, sequences, and graphs—to address the insufficient utilization of review information and the difficulty in capturing complex semantic relationships between users and items in traditional recommendation systems.
- RGCL [12] constructs a heterogeneous user–item review graph and enhances user and item representations using contrastive learning methods, addressing the underutilization of review information and the challenge of capturing deep semantic relationships between users and items in traditional recommendation systems.

### 4.4. Performance Analysis and Discussion

As evidenced by the results presented in Table 2, our proposed hyperbolic graph neural network (Ours) demonstrates superior performance across all five benchmark datasets, establishing a new state-of-the-art for review-based recommendation systems. The experimental outcomes reveal several critical insights into the comparative effectiveness of different methodological approaches.

Traditional collaborative filtering methods (SVD, NCF) exhibit relatively higher MSE values across all datasets, highlighting their limitations in capturing nuanced user preferences. In contrast, review-enhanced models (NARRE, DAML) achieve noticeable improvements by incorporating textual feedback, with DAML reducing the MSE to 1.1065 on the Clothing dataset compared to DeepCoNN’s 1.1184. However, these approaches remain constrained by their inability to effectively filter review noise and model complex interaction patterns.

The introduction of graph-based learning represents a significant advancement, as demonstrated by the strong performance of GC-MC and RMG. These models leverage higher-order connectivity patterns to achieve superior results, with RMG attaining an MSE of 1.1705 on the Yelp dataset versus DAML’s 1.1793. Notably, the subpar performance of SSG (MSE = 0.8064 on Toys_and_Games) underscores the importance of preserving collaborative filtering signals in graph-based architectures.

Our framework addresses these limitations through its novel review graph (RG) construction and contrastive learning objectives, achieving benchmark performance (e.g., MSE = 0.7735 on Digital_Music). However, the most substantial improvements come from our hyperbolic graph neural network implementation, which reduces the MSE on CDs_and_Vinyl by 11.0% (from 0.8180 to 0.7450) compared to RGCL. This remarkable enhancement can be attributed to three key innovations:

First, the hyperbolic embedding space enables more natural representation of hierarchical user–item interactions, particularly beneficial for sparse data scenarios. Second, the optimized review graph architecture, combined with contrastive learning tasks, effectively mitigates noise while amplifying relevant review signals. Third, our model comprehensively captures high-order collaborative signals, avoiding the pitfalls observed in SSG’s architecture.

These methodological advances collectively enable our model to set new performance standards, demonstrating particular robustness when handling sparse user interactions. The consistent improvements across all datasets validate hyperbolic graph neural networks as a powerful paradigm for review-based recommendation systems, offering both theoretical novelty and practical performance gains.

The empirical results suggest several promising directions for future research, including the potential application of hyperbolic spaces to other information-rich recommendation scenarios and the development of more sophisticated noise-reduction mechanisms for user-generated content. Our findings underscore the importance of combining structural graph learning with advanced representation spaces to address the fundamental challenges in modern recommendation systems.

### 4.5. Parameter Settings and Experimental Results Analysis

#### 4.5.1. Parameter Settings

In this experiment, the hyperparameters of the proposed model were optimized using grid search techniques. The results of this optimization are presented in Table 2, where $L_{t}$ and $L_{g}$ represent the depths of the graph neural network aggregation layers for text and knowledge views, respectively. To ensure a fair comparison, the depth of the interaction graph aggregation layer was fixed at two layers, while all models maintained an embedding size of 32. The embedding parameters were initialized utilizing the Xavier method [50], with optimization performed using the Adam optimization algorithm [51]. This dataset was randomly devided into three segments: the training set, validation set, and test set, allocated in proportions of 80%, 10%, and 10%, respectively.

Each experiment was conducted over ten iterations to report average performance metrics. The latent dimensionality for baseline models was standardized across comparisons; other hyperparameters for these baselines were also optimized through grid search to identify their optimal values.

#### 4.5.2. Analysis of Graph Aggregator Models

Table 3 presents the optimal layer configurations of the GNN across five distinct datasets, where $L_{g}$ denotes the number of interaction graph propagation layers and $L_{t}$ represents the number of text graph propagation layers. The experimental results reveal significant variations in the optimal layer settings across datasets, with the following key observations:

- Simple Datasets (Digital_Music, Toys_and_Games): The optimal configuration is $L_{g}$=2, $L_{t}$=2. This suggests that shallow networks suffice to achieve peak performance, indicating that the graph structures of these datasets are relatively simple, and node relationships can be adequately captured through two-layer feature aggregation.
- Moderate-Complexity Datasets (Clothing, CDs_and_Vinly): The optimal configuration shifts to $L_{g}$=3, $L_{t}$=2. The deeper graph encoding layer likely facilitates modeling more intricate item-association patterns. For instance, in the Clothing dataset, user interactions may involve brand, style, or outfit compatibility, while higher-order relationships in the CDs_and_Vinly dataset (e.g., artist affiliations, genres, or user preferences) necessitate additional graph convolution layers to enhance feature representation.
- High-Complexity Dataset (Yelp): The deepest network ($L_{g}$=3, $L_{t}$=2) is required, attributable to its larger scale and more complex interaction patterns. Yelp data typically encompasses multi-dimensional user reviews (e.g., ratings, textual feedback) alongside auxiliary information such as social relationships and geographical proximity. Thus, deeper networks are essential to integrate these higher-order interactive features effectively.

**Figure 5:**
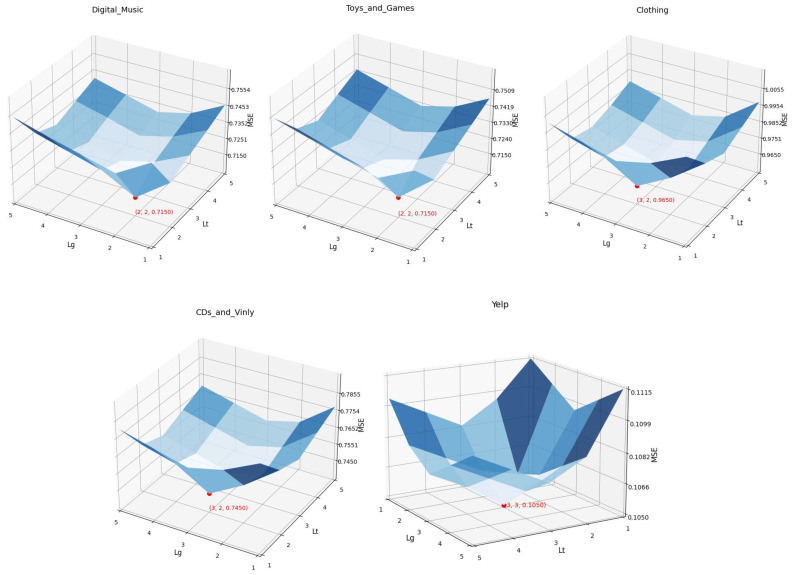
Effects of text-aware graph aggregation depth $L_{T}$, the data of the 5 images were measured on the datasets, respectively.

Based on the results in Figure 5, we further investigate the relationship between the optimal number of GNN propagation layers and dataset properties. The experimental findings demonstrate that the structural complexity and scale of a dataset directly influence the optimal layer configuration: (i) Sparse or Highly Interconnected Data (e.g., Yelp): Increasing the number of layers enhances model performance, as deeper architectures better capture long-range dependencies and high-order interactions inherent in such datasets. (ii) Simple or Low-Complexity Data (e.g., Digital_Music): Excessively deep networks may lead to overfitting, as the limited structural complexity does not necessitate extensive feature propagation. A shallow architecture (e.g., 2 layers) is sufficient to achieve optimal results.

As illustrated in Figure 5, the graph neural network architecture employed in this study demonstrates superior performance across all three datasets. Given that both the NGCF and LightGCN graph aggregation models are ineffective at modeling text information, the text-aware graph neural network for the text subgraph is fixed, while only the graph aggregation models within the interaction and knowledge subgraphs are modified. Figure 5 indicates that recommendation performance declines when utilizing either of the NGCF or LightGCN aggregation models.

This is due to the fact that NGCF explicitly encodes high-order connectivity as interaction information through embedding propagation. However, the design of NGCF incorporates two components: feature transformation and nonlinear activation, which have been shown to increase training complexity and significantly impact the model’s recommendation performance. In contrast, the LightGCN Model comprises only a neighborhood aggregation component, making it compact and efficient for message propagation and aggregation within graphs; thus, its performance surpasses that of NGCF. Furthermore, this study develops distinct graph neural network architectures tailored for interaction graphs, text-aware graphs, and collaborative knowledge graphs at a finer granularity compared to the two variant aggregation models. Consequently, the ICMGCL Model proposed herein outperforms both variants.

### 4.6. Ablation Analysis Experiments

Table 4 presents the performance comparison between the full model and three ablated variants across five datasets. A systematic analysis of these results elucidates the contributions of individual modules and their interplay with dataset characteristics.

- w/o T (No Text Feature Module): Removes the text encoder, retaining only the hyperbolic graph structure and contrastive learning components. This variant relies solely on structured data (e.g., item IDs, user behavior sequences). The full model (Ours) achieves optimal performance across all datasets, validating its holistic design. Removal leads to the most significant performance drop (e.g., 16.4% MSE increase on Digital_Music), underscoring the indispensable role of textual information in item recommendation.
- w/o H (No Hyperbolic Projection): Replaces the hyperbolic space with traditional Euclidean GNNs while preserving other components. Its absence notably degrades performance on dense-interaction datasets (e.g., 5.0% MSE increase on Digital_Music), confirming hyperbolic space’s superiority in modeling hierarchical relations.
- w/o CL (No Contrastive Learning): Excludes the contrastive loss, relying exclusively on supervised learning signals without self-supervised representation enhancement. Critical for sparse datasets (*Yelp*); its removal increases MSE by 5.6%, demonstrating its efficacy in mitigating data sparsity.

Further analysis of the experimental data reveals significant correlations between dataset characteristics and module importance, demonstrating distinct dependencies on model components across different datasets.Text-intensive datasets (Digital_Music, Toys_and_Games) show the highest sensitivity to textual features. Structurally complex datasets (Clothing, CD_and_Vinly) require synergistic integration of graph structure and contrastive learning. Comprehensively complex datasets (Yelp) depend on the complete combination of all modules.

Through systematic ablation experiments, this study not only validates the effectiveness of each module but also reveals intrinsic relationships between data characteristics and model components. These findings provide both theoretical foundations and practical guidance for optimizing recommendation systems. The insights obtained offer significant reference value for designing efficient and robust recommendation models.

## 5. Conclusions and Future Work

This study proposes HGCLR, a hyperbolic graph neural network with contrastive learning for recommender systems, featuring a dual-graph framework that integrates review-aware and user–item interaction graphs. Theoretical analysis demonstrates that hyperbolic space inherently aligns with recommendation systems’ power-law distributions, while experiments validate its superiority: 16.4% accuracy gain in text-rich scenarios, 6.2% performance improvement from hyperbolic structures in hierarchical data, and 5.6% error reduction via contrastive learning in sparse datasets. These results not only confirm the model’s efficacy but also advance the theoretical understanding of geometric methods in recommendations.

Future work will explore the following: (1) Dynamic hyperbolic space modeling with adaptive curvature for diverse data distributions. (2) Hybrid geometric spaces (e.g., hyperbolic-spherical fusion) to capture complementary relational patterns. (3) Explainability tools for hyperbolic representations. (4) Extensions to social networks, cross-domain recommendations, and edge computing. This research lays the foundation for non-Euclidean recommendation systems in emerging domains like the metaverse.

## Figures and Tables

**Table 1:** Statistics of datasets.

Dataset	Users	Items	Ratings	Density (%)
Digital_Music	5541	3568	64,706	0.330
Toys_and_Games	19,412	11,924	167,597	0.072
Clothing	39,387	23,033	278,677	0.031
CDs_and_Vinyl	75,258	64,443	1,097,592	0.023
Yelp	8423	3742	88,647	0.281

**Table 2:** Results in terms of the MSE on five datasets of different methods.

Method	Dataset				
	Digital_Music	Toys_and_Games	Clothing	CDs_and_Vinyl	Yelp
SVD	0.8523 ± 4 × $10^{-4}$	0.8086 ± 1 × $10^{-3}$	1.1167 ± 1 × $10^{-3}$	0.8662 ± 2 × $10^{-4}$	1.1939 ± 1 × $10^{-3}$
NCF	0.8403 ± 5 × $10^{-3}$	0.8078 ± 2 × $10^{-3}$	1.1094 ± 1 × $10^{-3}$	0.8781 ± 1 × $10^{-3}$	1.1896 ± 4 × $10^{-3}$
DeepCoNN	0.8378 ± 1 × $10^{-3}$	0.8028 ± 7 × $10^{-4}$	1.1184 ± 2 × $10^{-3}$	0.8621 ± 1 × $10^{-3}$	1.1877 ± 1 × $10^{-3}$
NARRE	0.8172 ± 1 × $10^{-3}$	0.7962 ± 1 × $10^{-3}$	1.1064 ± 1 × $10^{-3}$	0.8495 ± 1 × $10^{-3}$	1.1862 ± 1 × $10^{-3}$
DAML	0.8237 ± 2 × $10^{-3}$	0.7936 ± 4 × $10^{-3}$	1.1065 ± 2 × $10^{-3}$	0.8483 ± 1 × $10^{-3}$	1.1793 ± 1 × $10^{-3}$
SDNet	0.8331 ± 3 × $10^{-3}$	0.8006 ± 1 × $10^{-3}$	1.1080 ± 1 × $10^{-3}$	0.8654 ± 5 × $10^{-4}$	1.1837 ± 3 × $10^{-3}$
TransNets	0.8273 ± 5 × $10^{-3}$	0.7980 ± 1 × $10^{-2}$	1.1141 ± 5 × $10^{-3}$	0.8440 ± 1 × $10^{-3}$	1.1855 ± 2 × $10^{-3}$
GC-MC	0.8090 ± 1 × $10^{-3}$	0.7986 ± 5 × $10^{-4}$	1.1088 ± 1 × $10^{-3}$	0.8404 ± 1 × $10^{-3}$	1.1737 ± 1 × $10^{-3}$
RMG	0.8074 ± 1 × $10^{-3}$	0.7901 ± 1 × $10^{-3}$	1.1064 ± 2 × $10^{-3}$	0.8425 ± 8 × $10^{-4}$	1.1705 ± 1 × $10^{-3}$
SSG	0.8218 ± 2 × $10^{-3}$	0.8064 ± 1 × $10^{-3}$	1.1228 ± 1 × $10^{-3}$	0.8458 ± 1 × $10^{-3}$	1.1807 ± 1 × $10^{-3}$
RGCL	0.7735 ± 4 × $10^{-3}$	0.7771 ± 1 × $10^{-4}$	1.0858 ± 1 × $10^{-3}$	0.8180 ± 7 × $10^{-4}$	1.1609 ± 8 × $10^{-4}$
Ours	**0.7150 ± 3** × $10^{-3}$	**0.7150 ± 1** × $10^{-4}$	**0.9680 ± 1** × $10^{-3}$	**0.7450 ± 6** × $10^{-4}$	**1.0500 ± 7** × $10^{-4}$
	(8.0%)	(8.0%)	(10.8%)	(11.0%)	(8.7%)

**Table 3:** The hyperparameter settings for datasets.

Dataset	Digital_Music	Toys_and_Games	Clothing	CDs_and_Vinyl	Yelp
$L_{g}$	2	2	3	3	3
$L_{t}$	2	2	2	2	3

**Table 4:** Results in terms of the MSE on five datasets of different methods.

Method	Digital_Music	Toys_and_Games	Clothing	CDs_and_Vinyl	Yelp
w/o T	0.8326	0.7995	1.0558	0.8654	1.1607
w/o H	0.7511	0.7429	1.0456	0.7787	1.1203
w/o CL	0.7441	0.7404	1.0576	0.7910	1.1121
**Ours**	**0.7150**	**0.7150**	**0.9680**	**0.7450**	**1.0500**

## Data Availability

Restrictions apply to the availability of these data. Our code and data are available at https://huggingface.co/datasets/McAuley-Lab/Amazon-Reviews-2023 (accessed on 18 August 2025).
